# *Centella asiatica* and its caffeoylquinic acid and triterpene constituents increase dendritic arborization of mouse primary hippocampal neurons and improve age-related locomotion deficits in *Drosophila*

**DOI:** 10.3389/fragi.2024.1374905

**Published:** 2024-07-11

**Authors:** Karon Rowe, Nora E. Gray, Jonathan A. Zweig, Alexander Law, Natascha Techen, Claudia S. Maier, Amala Soumyanath, Doris Kretzschmar

**Affiliations:** ^1^ BENFRA Botanical Dietary Supplements Research Center, Oregon Health and Science University, Portland, OR, United States; ^2^ Oregon Institute of Occupational Health Sciences, Oregon Health and Science University, Portland, OR, United States; ^3^ Department of Neurology, Oregon Health and Science University, Portland, OR, United States; ^4^ National Center for Natural Products Research, University of Mississippi, Oxford, MS, United States; ^5^ Department of Chemistry, Oregon State University, Corvallis, OR, United States; ^6^ Linus Pauling Institute, Oregon State University, Corvallis, OR, United States

**Keywords:** asiatic pennywort, gotu kola, 1,5-dicaffeoylquinic acid, aging, fast phototaxis

## Abstract

**Introduction:**

*Centella asiatica* (CA) is known in Ayurvedic medicine as a rejuvenating herb with particular benefits in the nervous system. Two groups of specialized metabolites found in CA and purported to contribute to its beneficial effects are triterpenes (TTs) and caffeoylquinic acids (CQAs). In order to evaluate the role and interactions of TTs and CQAs in the effects of CA, we examined the neurotrophic effects of a water extract of CA (CAW) and combinations of its TT and CQA components in mouse primary hippocampal neurons *in vitro* and in *Drosophila melanogaster* flies *in vivo*.

**Methods:**

Primary hippocampal neurons were isolated from mouse embryos and exposed *in vitro* for 5 days to CAW (50 μg/mL), mixtures of TTs, CQAs or TT + CQA components or to 4 TTs or 8 individual CQA compounds of CAW. Dendritic arborization was evaluated using Sholl analysis. *Drosophila* flies were aged to 28 days and treated for 2 weeks with CAW (10 mg/mL) in the food, mixtures of TTs, CQAs or TT + CQA and individual TT and CQA compounds. TTs and CQAs were tested at concentrations matching their levels in the CAW treatment used. After 2 weeks of treatment, *Drosophila* aged 42 days were evaluated for phototaxis responses.

**Results:**

In mouse primary hippocampal neurons, CAW (50 μg/mL), the TT mix, CQA mix, all individual TTs and most CQAs significantly increased dendritic arborization to greater than control levels. However, the TT + CQA combination significantly decreased dendritic arborization. In *Drosophila,* a marked age-related decline in fast phototaxis response was observed in both males and females over a 60 days period. However, resilience to this decline was afforded in both male and female flies by treatment from 28 days onwards with CAW (10 mg/mL), or equivalent concentrations of mixed TTs, mixed CQAs and a TT + CQA mix. Of all the individual compounds, only 1,5-diCQA slowed age-related decline in phototaxis in male and female flies.

**Discussion:**

This study confirmed the ability of CAW to increase mouse neuronal dendritic arborization, and to provide resilience to age-related neurological decline in *Drosophila*. The TT and CQA components both contribute to these effects but do not have a synergistic effect. While individual TTs and most individual CQAs increased dendritic arborization at CAW equivalent concentrations, in the *Drosophila* model, only 1,5-diCQA was able to slow down the age-related decline in phototaxis. This suggests that combinations (or potentially higher concentrations) of the other compounds are needed to provide resilience in this model.

## Introduction

Due to increasing life expectancy, the population of elderly is increasing worldwide. It is predicted that the current number of people over 65 years will have doubled by 2050 (He et al., 2016). However, aging is associated with various health threats, including reduced locomotion, cognitive decline, and the development of chronical medical conditions like hypertension or diabetes (Fried et al., 2004). The increase in the elderly population has therefore generated a surge of interest in interventions that could improve quality of life and minimize or delay age-related deficits and diseases. This includes complementary and alternative medicine that have traditionally been used to improve health particularly during aging. Consequently, botanical supplements that profess to have multiple resilience promoting effects have become quite common and mainstream (Nascimento et al., 2016; Ward and Pasinetti, 2016).

*Centella asiatica* (L.) Urban (CA) is one of the botanicals used to promote healthy neurological aging (Gray et al., 2018a). CA is an edible leafy green belonging to the Apiaceae family commonly found in India and other Asian countries, growing in moist environments. CA, also known as Indian pennywort, Asiatic pennywort, or Gotu Kola, has been used in traditional Ayurvedic and Chinese medicine for centuries for its wound healing and immunomodulatory effects, cardio-protective activity, and neurocognitive benefits (Gohil et al., 2010; Chandrika et al., 2015; Gray et al., 2018a). CA has been shown to improve cognition in aged mice (Gray et al., 2016; Gray et al., 2018b; Zweig et al., 2021a) and although clinical studies did not support a general improvement of learning and memory, CA did improve working memory in a clinical study with healthy elderly (Wattanathorn et al., 2008a; Puttarak et al., 2017). CA was also shown to ameliorate the cognitive deficits in mouse models of Alzheimer’s disease (Tiwari et al., 2008; Soumyanath et al., 2012; Zweig et al., 2021b) and in a clinical trial of patients with Mild Cognitive Impairment (Tiwari et al., 2008; Soumyanath et al., 2012; Zweig et al., 2021b). Furthermore, it has been described that CA can decrease blood pressure in elderly with hypertension (Astutik and Fauzia Zuhroh, 2021) and increase specific aspects of physical performance in healthy elderly individuals (Mato et al., 2011a). CA consumption is considered quite safe with only rare and mild side effects like headaches or gastrointestinal discomfort (Gohil et al., 2010; Biswas et al., 2021; Wright et al., 2022). This is an important aspect, considering that it might be consumed regularly over months, years or even decades to promote healthy aging.

To investigate a role for CA in promoting neuronal health and to identify the active compounds within CA mediating these effects, we combined *in vitro* cell culture models with the *in vivo Drosophila melanogaster* model. We previously showed that a water extract of CA (CAW) can protect neuroblastoma cells from oxidative stress and Aβ-induced lethality (Gray et al., 2014; Yoon et al., 2023). It can also improve mitochondrial function and content in wild type mouse primary hippocampal neurons (Gray et al., 2017). CAW also improved cognitive function in mouse models of aging and Alzheimer’s disease (Gray et al., 2016; Gray et al., 2018b; Zweig et al., 2021a). We therefore now used mouse primary hippocampal neurons to determine whether CAW and CA compounds, namely, CQAs and TTs, can improve neuronal outgrowth and complexity. In addition, we used *Drosophila* as an *in vivo* model to determine to what extend CAW, CQAs, and TTs can provide resilience against age-related changes in activity and locomotion.

## Materials and methods

### Plant material–source and authentication

Dried CA plant material was purchased from international vendors (Organic India) and supplied for the project by Oregon’s Wild Harvest (Redmond, Oregon, United States). A voucher sample of the material (designated “BEN-CA6”) is maintained at Oregon Health & Science University (OHSU), and a second voucher sample (OSC-V-265416) has been deposited at the Herbarium at Oregon State University (Corvallis, OR). BEN-CA6 was authenticated as CA by DNA barcoding. DNA was extracted from the sample using the DNeasy Mini Kit (Qiagen) according to the manufacturer’s instructions. Primers to amplify the ITS genomic region were designed using Primer BLAST using already published (Ye et al., 2012) *Centella* ssp ITS sequences. Designed primers consisted of a 5′ adapter sequence (pJet) facilitating direct sequencing with universal pJet primers of single PCR products. Primers (Table 1) were synthesized by Integrated DNA Technologies (Coralville). Extracted DNA was then diluted 1:10 and the ITS region amplified with the designed primers. Amplification consisted of two rounds of PCR. The first PCR consisted of a 25-μL reaction mixture containing 2 μL of a 1:10 DNA dilution, 1 × PCR reaction buffer, 0.2 mM dNTP mixture, 0.2 μM of each forward and reverse primers CentITSF and CentITSR, 1.5mM MgCl2, and 1 U of Platinum Taq DNA Polymerase (Invitrogen). The first PCR program consisted of an initial denaturation step at 94°C for 3 min, followed by 10 cycles at 94°C for 80 s, 58°C for 25 s and 72°C for 1 min. The second PCR consisted of a 25-μL reaction mixture as mentioned above using 1 μL of the first PCR as a template and pJet forward/reverse primers. The second PCR program consisted of 35 cycles with denaturation at 94°C for 30 s, 65°C for 20 s, and 72°C for 1min, with a final extension at 72°C for 3 min. After amplification, 10 µL were analyzed by electrophoresis on 1.5% borate agarose gel and visualized under UV light. PCR products were compared to the molecular size standard 1 kb plus DNA ladder (Invitrogen). Resulting PCR products were cloned into Vector pCR4-TOPO TA (Invitrogen) and transferred into *E. coli* according to the manufacturer’s instructions. Eight transformants per cloning event were subjected to colony PCR using M13F and M13R primers. The PCR products of four transformants per DNA were sequenced with M13 forward and reverse primers at Genewiz/Azenta. Derived sequences were trimmed and subjected to homology search against the NCBI nucleotide database using BLAST available in Geneious Prime 2023.2.1 (Biomatters. LTD) [ (Altschul et al., 1990)]. Additionally, a neighbor-joining consensus tree was built with the Tamura-Nei Distance model without an outgroup, bootstrap 500 replicates, available in Geneious Prime 2023.2.1.

**TABLE 1:** Primers used to amplify the ITS genomic region.

Primer name	Sequence with pJet adapter (5′ end)
CentITSF	CGA​CTC​ACT​ATA​GGG​AGA​GCG​GC-CGC​GAA​CAC​GTA​AAG​CAA​CA
CentITSR	AAG​AAC​ATC​GAT​TTT​CCA​TGG​CAG-TGG​CAA​CAG​GGT​CGC​A
PJET1-2F	CGA​CTC​ACT​ATA​GGG​AGA​GCG​GC
PJET1-2R	AAG​AAC​ATC​GAT​TTT​CCA​TGG​CAG
M13F (-21)	TGTAAAACGACGGCCAGT
M13R	CAGGAAACAGCTATGAC

### Plant extract

The preparation of the CA water extract used in this study (BEN-CAW-7) has been previously described in detail (Holvoet et al., 2023a; Yang et al., 2023a). BEN-CA-6 plant material (4 kg) was extracted by boiling in deionized water (50 L) for 90 min. The extract was filtered, frozen and lyophilized in three batches to yield dried extracts BEN-CAW-7, 8 and 9 with a total extract weight of 820g (20.5% of the starting dried plant material). Voucher samples of BEN-CAW 7, 8 and 9 are stored at OHSU. CAW-7 was used in the present study and will be referred to as “CAW” in the rest of this article.

### Triterpenes and caffeoylquinic acids in CAW

Four purified triterpenes (TTs) and eight caffeoylquinic acids (CQAs) known to occur in CAW (RdS et al., 2022) were purchased from commercial sources and their identity verified by us as previously described (Holvoet et al., 2023b). The content of these compounds in the CAW batch used in this study was determined by liquid chromatography coupled to multiple reaction monitoring mass spectrometry (LC-MRM-MS) as previously reported by us (Holvoet et al., 2023b; Yang et al., 2023b). Content (% w/w) of the selected TT and CQA compounds in CAW were asiatic acid, 0.042%; madecassic acid, 0.075%; asiaticoside, 1.475%; madecassoside 3.589%; chlorogenic acid (3-CQA), 0.750%, cryptochlorogenic acid (4-CQA), 0.298%; neochlorogenic acid (5 CQA), 0.339%; 1,3-diCQA, 0.258%; 1,5- diCQA, 0.389%; Isochlorogenic acid A (3,5- diCQA), 0.177%; Isochlorogenic acid B (3,4- diCQA), 0.229%, and Isochlorogenic acid C (4,5- diCQA), 0.195%. These values were used to make up solutions of individual compounds or mixtures equivalent to their content in the concentrations of CAW tested in primary neuron and *Drosophila* experiments.

### Mouse primary hippocampal neuron culture

Mice were housed in an AALAC certified facility and maintained in a climate-controlled environment with a 12-h light/12-h dark cycle and fed a Pico Lab Rodent Diet 5LOD (LabDiets, St. Louis, MO, United States). Diet and water were supplied *ad libitum*. All procedures were conducted in accordance with the NIH Guidelines for the Care and Use of Laboratory Animals and were approved by the institutional Animal Care and Use Committee of OHSU.

C57BL6 breeding pairs were acquired from Jackson Laboratories. Hippocampal neurons were isolated from C56BL6 embryonic mice, based on the methods of (Kaech and Banker, 2006). Embryos were harvested at 18 days of gestation from anesthetized females. Hippocampi were dissected, gently minced, trypsinized, and triturated to generate suspensions of dispersed neurons. Neurons were plated at a density of 130,000 cells in 60 mm dishes in MEM medium (GIBCO/Life Technologies, Waltham, MA, United States), 5% FBS (Atlanta Biologicals, Flowery Branch, GA, United States), 1× Anti-Anti (GIBCO/Life Technologies) and 0.6% glucose (Sigma-Aldrich, St. Louis, MO, United States), each dish containing 3 poly-l-lysine-coated nitric acid treated glass coverslips with paraffin wax spacers. After 3 h, the coverslips were flipped into 60 mm dishes containing mouse neural stem cell-derived glial cells (provided by Dr. Gary Banker, Jungers Center, OHSU) and maintained in 6 mL Neurobasal Medium supplemented with 1× GlutaMAX (GIBCO/Life Technologies), 1× Anti-Anti (GIBCO/Life Technologies) and 1× GS21 neural supplement (ThermoFisher, Waltham, MA, United States). Dishes were fed every week by removing 1 mL of the culture medium and adding 1 mL fresh Neurobasal medium that included GlutaMAX, Anti-Anti and Neuronal Culture Medium Supplement, with the first feed at 5 days *in vitro* (DIV) containing 6 μM (1 μM final) cytosine β-D-arabinofuranoside hydrochloride (AraC; Sigma-Aldrich).

After 14 days *in vitro* (DIV) cells were treated with either CAW (50 μg/mL), TT mix (containing asiatic acid, madecassic acid, asiaticoside and madecassoside), CQA mix (containing 3-CQA, 4-CQA, 5-CQA, 1,3-diCQA, 1,5-diCQA, isochlorogenic acid A, isochlorogenic acid B and isochlorogenic acid C), TT + CQA mix, individual TTs or individual CQAs. The vehicle used to resuspend the compounds was 25% methanol and treatment stocks were prepared at 100x so the final concentration of methanol in the cell culture media was 0.25%. The concentrations of TTs and CQAs tested individually or in mixtures were equivalent to their relative abundance in CAW (50 μg/mL). Results were compared to vehicle-treated neurons.

### Assessment of dendritic complexity

At 19 DIV coverslips were fixed in 4% paraformaldehyde and stained with Anti-MAP2B (Sigma-Aldrich #M4403; 3.3 μg/mL) and Goat anti-mouse IgG1-Cy3 (Jackson ImmunoResearch #115-165-205; 1.5 μg/mL). Immunostained neurons were imaged with a Zeiss ApoTome2 microscope and blinded Sholl analyses were performed to assess dendritic complexity using the Fiji platform. Analysis consisted of quantifying the number of intersections of dendritic processes with concentric circles drawn at 10 μm intervals from the cell body out to 300 μm. Plotting the number those intersections as a function of distance from the cell body generates a curve and results are represented as area under the curve. Thirty isolated, non-overlapping cells were analyzed per coverslip. Arborization data was pooled across 3 independent experiments (3–5 coverslips per treatment condition in each experiment) providing between 180 and 270 cells per condition over 3 experiments (60-90 cells per condition in each experiment).

Statistical analyses were done using GraphPad (v.5 for windows, San Diego CA, United States). ANOVAs were used to compare multiple groups with Tukey’s *post hoc* pairwise testing. Significance levels are indicated by asterisk with *p < 0.05, **p < 0.01, ***p < 0.001.

### Fly stock and treatment

The *D. melanogaster* wild-type Canton S (CS) stock was originally provided by M. Heisenberg, University of Würzburg, Germany. Flies were maintained on standard *Drosophila* food in a 12:12 h light and dark cycle at 24°C. For treatment, newly eclosed flies were collected daily and aged on standard food for 4 weeks with fresh food provided every week. Flies were then divided into a control and a treatment group. The first treatment paradigm involved mixing CAW into standard fly food at a final concentration of 10 mg/mL (from a stock solution of 100 mg/mL CAW in water) whereas the food for the control group was mixed with the same amount of water. A second treatment paradigm was to provide the supplement (CAW 10 mg/mL) in 10% yeast paste (Fleischmann’s Dry Yeast), whereby the yeast paste was applied to a tissue on the surface of standard food. Yeast paste with water added instead of the CAW supplement was used for the controls.

Application by yeast paste was also used when testing individual compounds and compound mixes. If not stated otherwise, the concentration of compounds was equivalent to their levels found in CAW as previously determined (Yang et al., 2023a; Holvoet et al., 2023b). For treatment of *Drosophila* a 10x concentrated stock solution of the compound or compounds was prepared in ethanol and diluted 1:10 in yeast paste. Control yeast paste for these experiments was prepared by adding ethanol instead of supplement (final concentration 10% ethanol). The flies were given supplemented or control food for two more weeks, changing the food once, before being tested when 6 weeks old.

### Fast phototaxis assays

Fast phototaxis assays were conducted as previously described (Bolkan et al., 2012; Dutta et al., 2016). Briefly, flies were transferred to the apparatus in groups of 10–15 flies, shaken to the bottom of the tubes and allowed to transition towards the light in 5 consecutive runs, each lasting 6s. Flies were then scored based on the tube they were contained in at the end of the final run. A detailed description of the experimental conditions can be found in (Strauss and Heisenberg, 1993). Statistical analyses were done using GraphPad (v.5 for windows, San Diego CA, United States). Normal distribution was addressed with the D’Agostino and Pearson omnibus test and due to the nonparametric distribution, Mann-Whitney tests were used to determine significance between two samples and Kruskal–Wallis ANOVA (with built-in Bonferroni *post hoc*) were used when comparing multiple groups. Significance levels are indicated by asterisks with *p < 0.05, **p < 0.01, ***p < 0.001.

## Results

### Verification of plant material as CA

The genus *Centella* contains 53 species and we first wanted to confirm that the provided plant material was *C. asiatica*. We therefore sequenced two samples derived from the provided plant material; BEN-CA6-A and BEN-CA6-B, and found that BEN-CA6-A was 92.4% identical to BEN-CA6-B. In the NCBI database ITS sequences from only three *Centella* species were available which were used for comparison. The BLAST homology search of BEN-CA6-A resulted in a sequence identity to *C*. *asiatica* of 98.7%–99.3%, to *Centella capensis* 91.4%, and to *Centella montana* 76.3%. The sequence identity of BEN-CA6-B to *C*. *asiatica* was 91.1%–91.8%, to *C. capensis* 86.8%, and to *C. montana* 71.1% (Supplementary Figure S1). We therefore concluded that the plant material was *C*. *asiatica*.

### CAW, and CQA and TT mixes increase dendritic arborization in mouse primary neurons, but the combination of CQAs and TTs does not

To evaluate the effects of CAW and its constituent compounds on neuronal complexity, we measured dendritic arborization in an *in vitro* system. Primary, embryonic, hippocampal neurons isolated from C57BL/6 mice were grown in culture on a glial feeder layer for a total of 19 days with the neurons exposed to treatments during the last 5 days.

**FIGURE 1:**
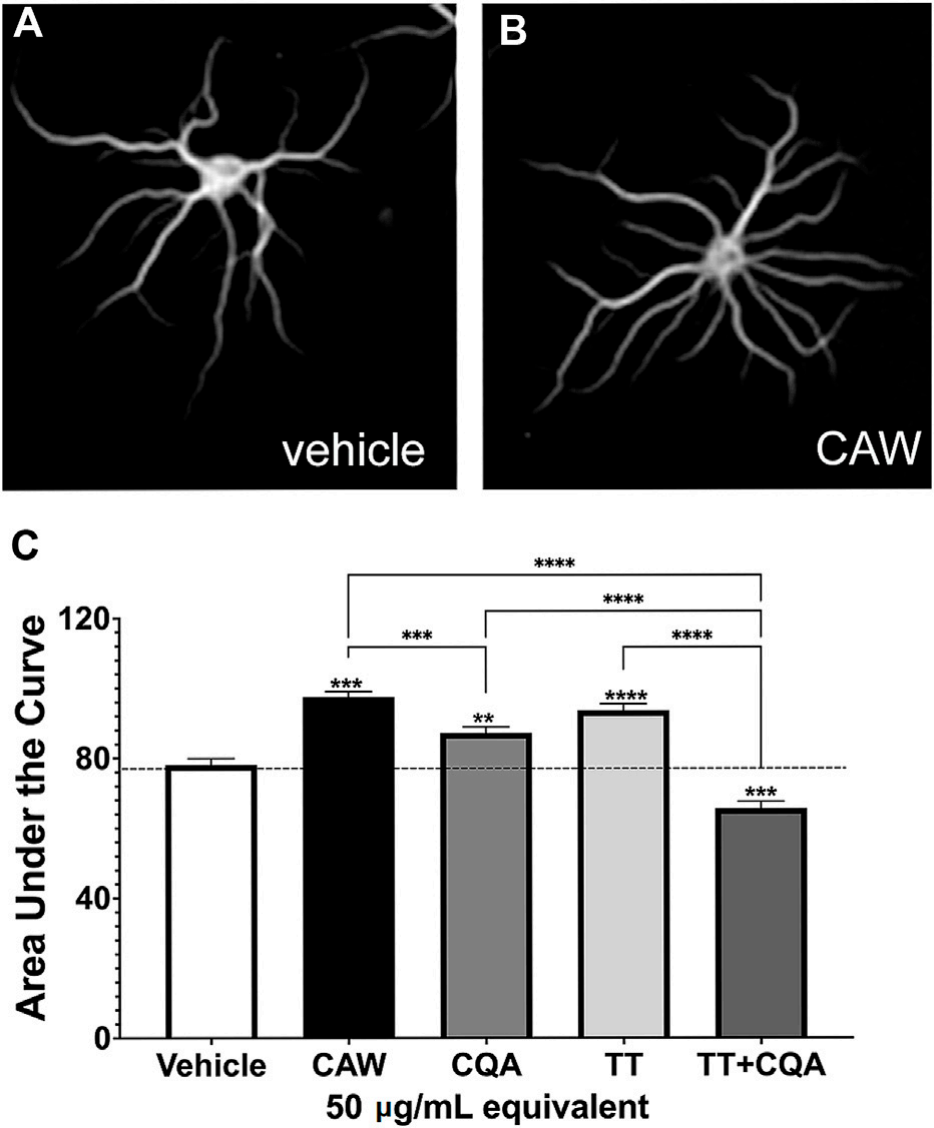
CAW treatment increases dendritic arborization in mouse primary hippocampal neurons as does treatment with groups of its constituent compounds. Mouse embryonic hippocampal neurons were cultured for a total of 19 days. During the final 5 days of culture they were treated with the vehicle **(A)** or exposed to treatment with 50 μg/mL CAW **(B)**, CQAs, TTs or the combination of CQA + TT at concentrations equivalent to their presence in CAW (50 μg/mL) (for representative images see Supplementary Figure S2A). **(C)** CAW, CQA and TT treatment all increased arborization while the combination of CQA + TT reduced arborization. Between 180 and 270 cells were analyzed per treatment condition. Error bars represent SEMs. An ANOVA test was used to determine significance between control and treatment groups with Tukey *post hoc* pairwise testing. * = *p* < 0.05, ** = *p* < 0.01, *** = *p* < 0.001.

We previously showed that treating hippocampal neurons with CAW (50 μg/mL) increased neuronal complexity (Gray et al., 2017). The current batch of CAW at 50 μg/mL also increased the dendritic complexity (Figure 1B) compared to vehicle treated neurons (Figure 1A). To quantify this, we performed a Scholl analysis and measured the area under the curve generated by these values (Figure 1C). Treatment with CQA mix and the TT mix, at concentrations equivalent to their relative abundance in CAW (50 μg/mL), each also increased arborization although the magnitude of increase was less with CQA treatment than was seen with CAW treatment. Surprisingly, the combination of TT and CQA mixes resulted in reduced arborization relative to vehicle treated neurons (Figure 1C). Representative images of the neurons treated with the compound mixes are shown in Supplementary Figure S2A.

### Individual TT and CQA compounds increase dendritic arborization in mouse primary neurons

**FIGURE 2:**
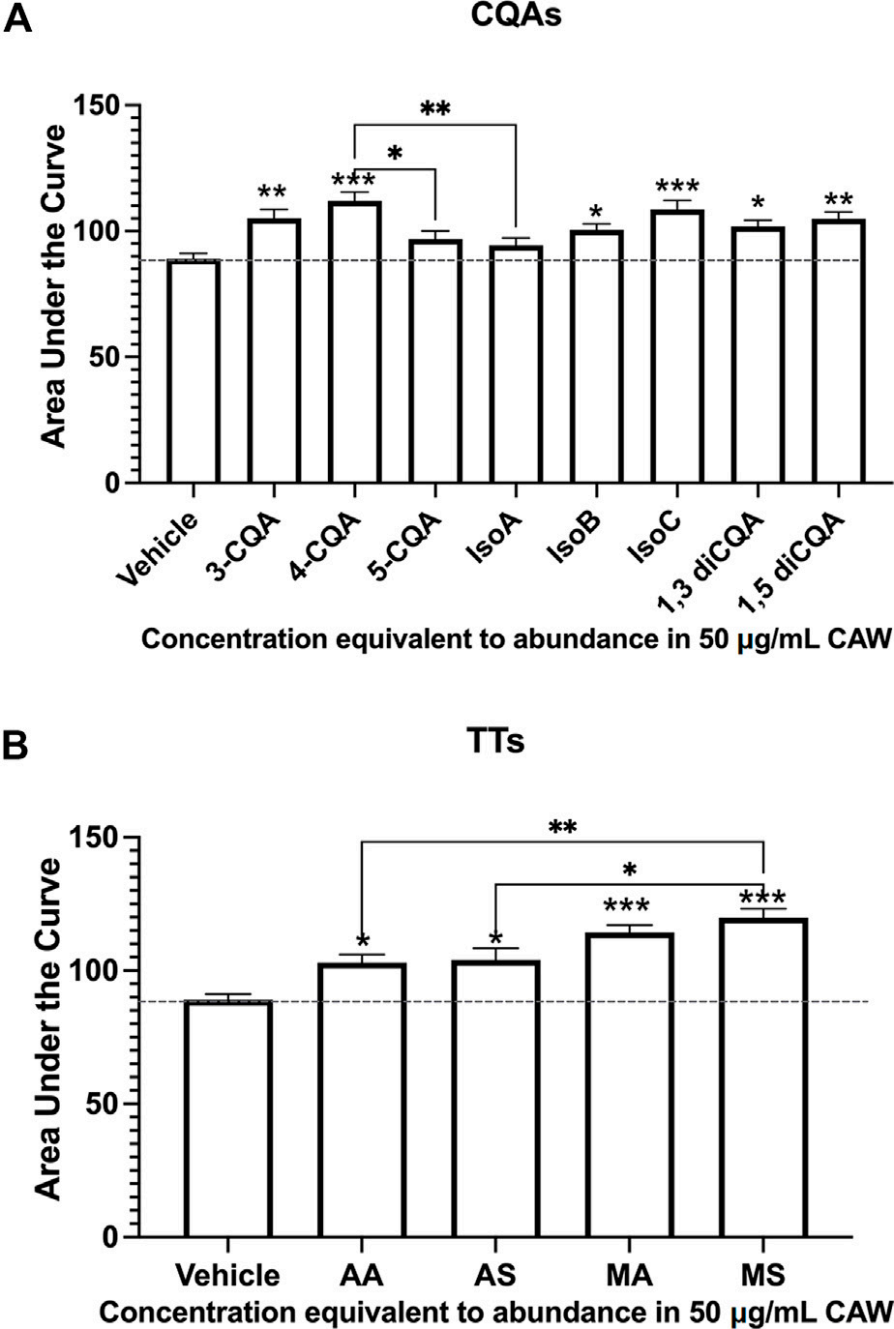
Individual constituent compounds from CAW also increase arborization in mouse hippocampal neurons. Mouse embryonic hippocampal neurons were again cultured for a total of 19 days, during the final of which they were exposed to treatment of individual CQA **(A)** or TT **(B)** compounds at concentrations equivalent to their presence in CAW (50 μg/mL). **(A)** All CQAs with the exception of 5-CQA (neochlorogenic acid) and IsoA (3,5-diCQA; isochlorogenic acid) increased arborization. **(B)** Similarly, all individual TTs increased arborization. Between 180 and 270 cells were analyzed per treatment condition (for representative images see Supplementary Figures S2B, C). Error bars represent SEMs. An ANOVA test was used to determine significance between controls and treatment groups with Tukey *post hoc* pairwise testing. * = *p* < 0.05, ** = *p* < 0.01, *** = *p* < 0.001.

We went on to test the effects of the individual CQA and TT compounds present in the mixes on dendritic arborization in the same primary hippocampal neuron system, again, at concentrations equivalent to their abundance in CAW (50 mg/mL). We found that while six out of the 8 individual CQAs tested increased arborization above what was seen with vehicle treated neurons, 5-CQA (neochlorogenic acid) and isochlorogenic acid A (IsoA) did not affect arborization (Figure 2A). In contrast, treatment with each of the 4 TT compounds did result in significant increases in arborization, with MS eliciting a greater magnitude of change than the other TT compounds (Figure 2B). Representative images of the neurons treated with the single compounds are shown in Supplementary Figures S1A, C.

### CAW supplementation ameliorates the age-related decline in fast phototaxis in *Drosophila*

**FIGURE 3:**
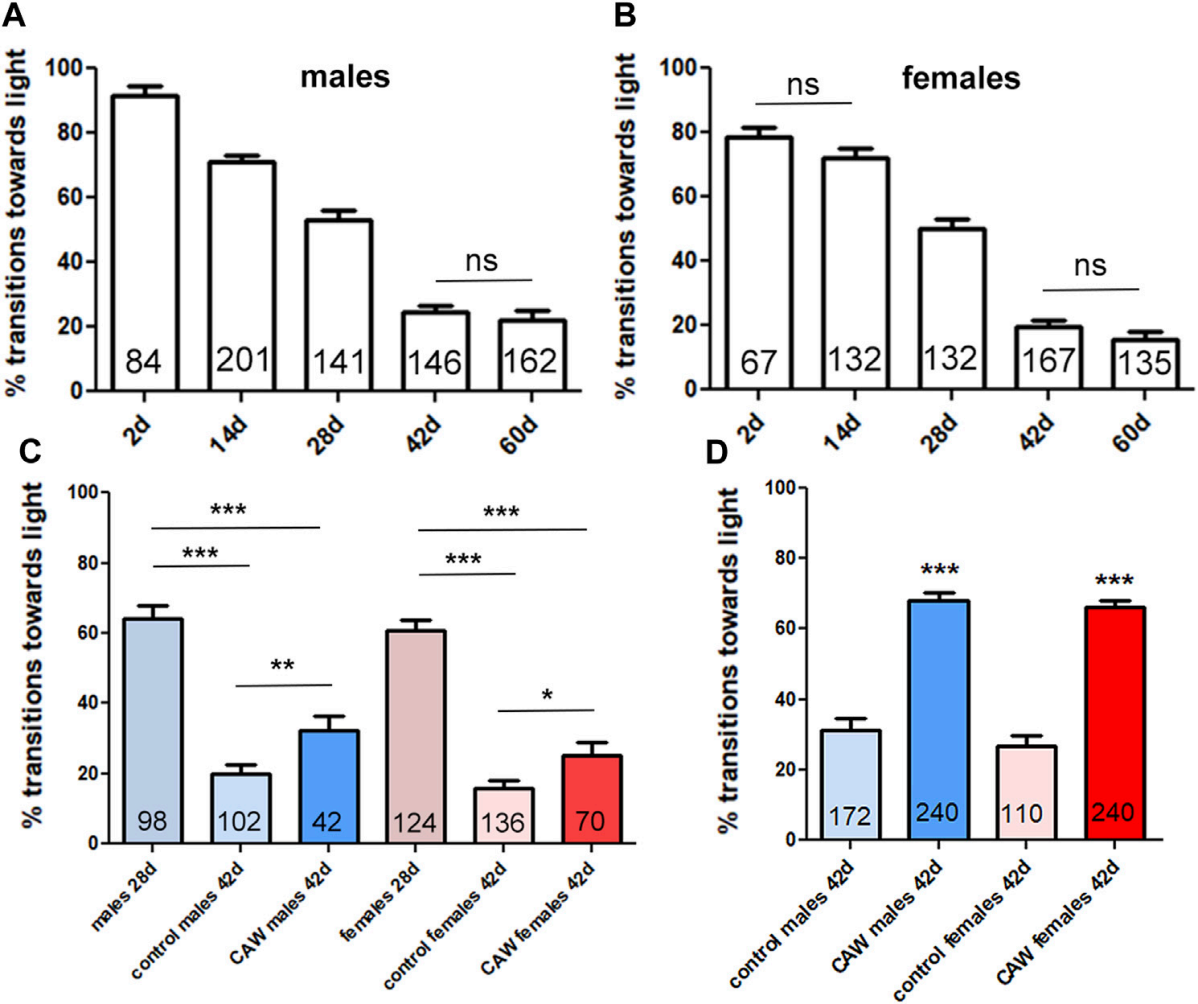
CAW provides resilience to the age-dependent decline in fast phototaxis. *Drosophila* males **(A)** and females **(B)** show a progressive decrease in performance with age. A Kruskal–Wallis tests for comparing multiple groups with Dunn’s post-tests to compare all groups showed significance for all comparisons besides the pairs indicated by ns. **(C)** Providing CAW (10 mg/mL) in the food for 2 weeks before testing significantly improves the performance in 42 days old males and females. Mann-Whitney tests were used to determine significance between controls and treatment groups at 42 days. Kruskal–Wallis tests for comparing multiple groups with Dunn’s post-test to compare selected pairs was used to determine differences between 28days and 42 days old flies. **(D)** Adding CAW (10 mg/mL) to yeast paste showed a bigger improvement, with increasing the mean transitions towards light from 31% to 68% in males and 27%–66% in females. Mann-Whitney tests were used to determine significance between controls and treatment groups. Fast phototaxis was performed with flies in groups of 10–15 flies and separated by experimental group and sex. The number of flies tested is indicated in the bars. Error bars represent SEMs. * = *p* < 0.05, ** = *p* < 0.01, *** = *p* < 0.001.

Besides improving memory, CA has also been described to improve physical performance in the elderly (Mato et al., 2011b)*. Drosophila*, like humans, show cognitive decline and behavioral changes when aged, including a reduction in locomotion (Iliadi and Boulianne, 2010). We therefore used this model to address whether CA provides resilience against age-related phenotypes using the *Drosophila in vivo* model. Using the fast phototaxis assay, which tests locomotion and the ability of the flies to detect and orient towards light, we first showed that the performance in this test is continuously reduced with age in male and female flies (Figures 3A, B). We also performed this experiment to determine an appropriate time point for treatment that would allow us to detect effects of CAW during aging. As shown in Figures 3A, B, both sexes show a continuous decline with age up to 42 days (only the difference between 2days and 14 days old females and between 42days and 60 days old male and female flies did not reach significance). We picked 28 days as an age to start treatment because these are middle-aged flies that show a significant decline with age at mid-age (28 days) that progresses further with continuing aging. To determine whether supplementation with CAW can ameliorate further decline in performance when given at mid-age, we kept the flies for 28 days on regular food. We then mixed the food with CAW and kept the flies on it for two more weeks before testing them in the phototaxis assay. We previously found that a dose of CAW 10 mg/mL in the food has beneficial effects in a *Drosophila* model of oxidative stress (Cabey et al., 2022) and we therefore used this concentration in the present experiment to explore effects on healthy aging. When comparing the performance of control flies and flies treated from day 28 to day 42 with CAW, we found a significant improvement in both males and females (Figure 3C). This shows that CAW supplementation has a beneficial effect even when starting treatment at middle age. We also included untreated 28 days old flies to compare the decline in performance from 28 days old to 42 days old in untreated and CAW treated flies. While both groups showed a significant decrease from 28 days old to 42 days old, the decline in untreated males was 69% whereas it was only 50% in CAW treated males. A similar protection was seen in females, with a 74% reduction in untreated females between day 28 and day 42, compared, to a 59% reduction in CAW treated females (Figure 3C). We also tested a second treatment paradigm by giving the CAW in addition to the regular food by mixing it at 10 mg/mL in 10% yeast paste applied on top of the food. Yeast is a preferred food source for *Drosophila* that influences adult body weight and survival (Günther and Goddard, 2019). Correlating with the published positive effect on survival, providing the yeast paste did improved the mean performance in untreated flies; in males from 20% to 31% and in females from 16% to 27%. Providing CAW in yeast paste further improved performance to 68% in males and 66% in females (Figure 3D). Together this shows that both treatment paradigms provide resilience against the age-related decline in performance, even when treatment is started at middle age.

To confirm an effect of CAW treatment in another behavioral test, we performed a negative geotaxis test. This test does not require a stimulus and is based on the innate behavior of *Drosophila* to run upwards in a vertical tube. Using the same treatment paradigm as for the fast phototaxis, we tested 42 days old flies treated for 2 weeks before being tested. Again, we found that CAW supplementation improved the behavior in males and females (Supplementary Figure S3), confirming a beneficial effect of CAW on the fitness of aged flies.

### CQAs and TTs are active compounds in CAW but they do not have a synergistic effects

**FIGURE 4:**
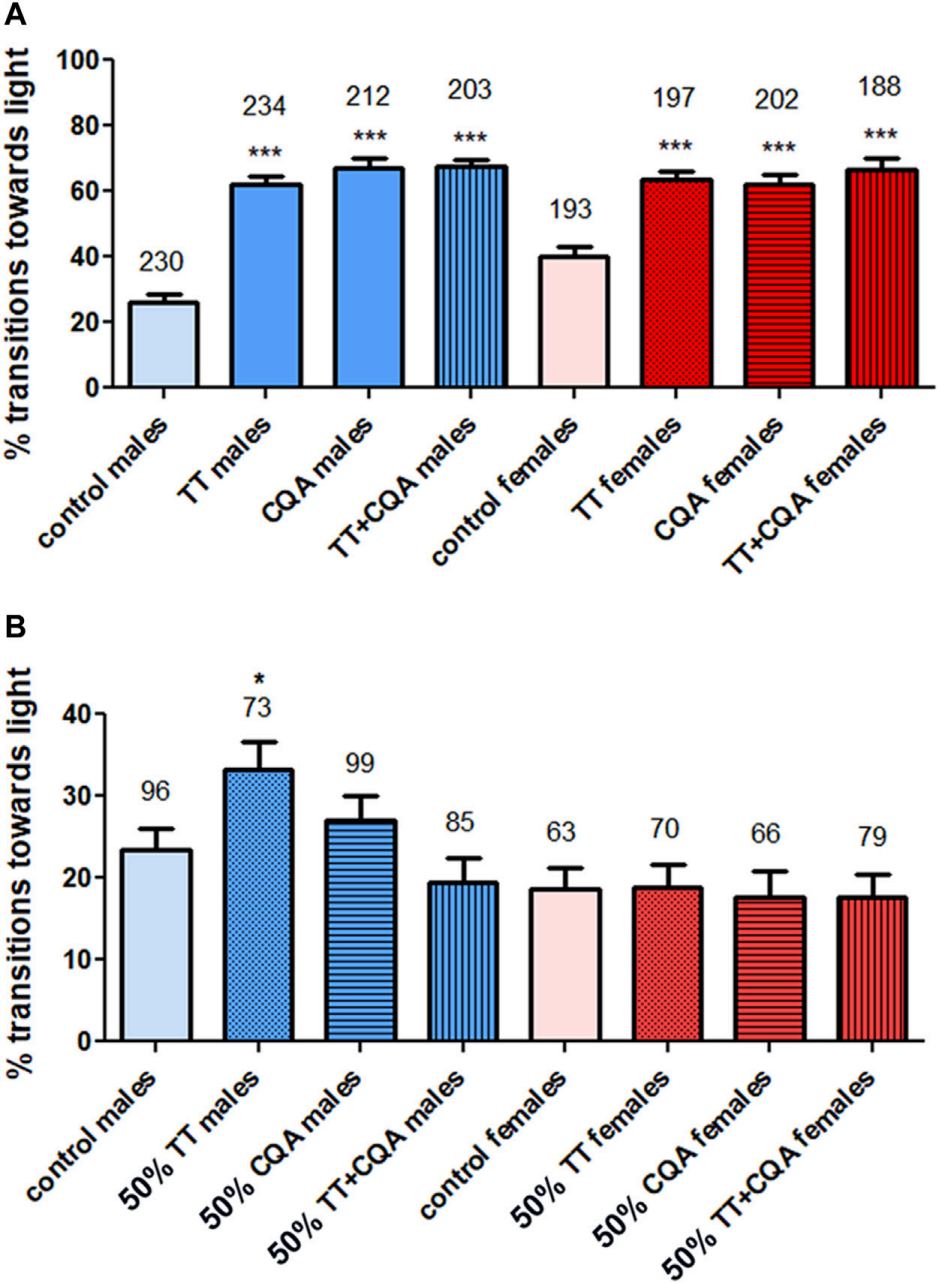
CQA and TT mixes are protective but combining them does not improve the efficacy. **(A)** Treating flies with a mix of CQAs or TTs at concentrations equivalent to 10 mg/mL CAW provides resilience to the decline in fast phototaxis in 42 days old males and females. Combining both of these mixes did not further improve performance. **(B)** Decreasing the concentration of the compounds in the mixes to 50% of the levels found in 10 mg/mL CAW prevented significant effects on phototaxis. An exception was the TT mix which was still protective in males at this lower concentration. Combining the lower concentration CQA and TT mix was also not protective, either in males or females. Fast phototaxis was performed with flies in groups of 10–15 flies and separated by experimental group and sex. The number of flies tested is indicated above the bars. Error bars represent SEMs. Kruskal–Wallis tests for comparing multiple groups with post Dunn’s post-tests to compare pairs were used to determine significances between control and treatment groups. * = *p* < 0.05, *** = *p* < 0.001.

Next, we tested whether the biological activity to provide resilience against the decline in fast phototaxis is mediated by CQAs or TTs. Due to the stronger effect when CAW was given in yeast paste, and the limited quantities of purified single compounds available, we tested CQAs and TTs only given in yeast paste. First, we tested mixes of CQAs and TTs containing the compounds in concentrations equivalent to CAW (10 mg/mL). We previously showed that the levels of these compounds in CAW are not affected by the preparation of the food and are stable for the 7 days the flies are kept on a food vial (Holvoet et al., 2023a). The compounds and compound mixes were prepared in ethanol as 10x stock solutions and therefore the control flies also obtained 10% ethanol in yeast paste. Both, the CQA mix and the TT mix significantly improved the behavior in males and females when given for 2 weeks to 28 days old flies (Figure 4A). Furthermore, both mixes had a similarly protective effect as CAW with 62% for the TT mix and 67% for CQA mix in males (CAW 68%) and 63% for the TTs and 62% for the CQAs in females (CAW 66%). We also tested whether combining both mixes (TT + CQA) further improved the behavior but this was not the case. We then asked whether the lack of a synergistic effect when the CQAs and TTs were combined was due to a “ceiling effect” due to the single mixes already being similarly effective as CAW. To address this, we reduced the concentration of the mixes by 50%. This resulted in a loss of protection by any of the mixes with the exception of the TT mix in males (Figure 4B). Furthermore, we still did not see a positive effect of combining the mixes. In fact, adding the CQAs prevented the protection seen with the TTs.

**FIGURE 5:**
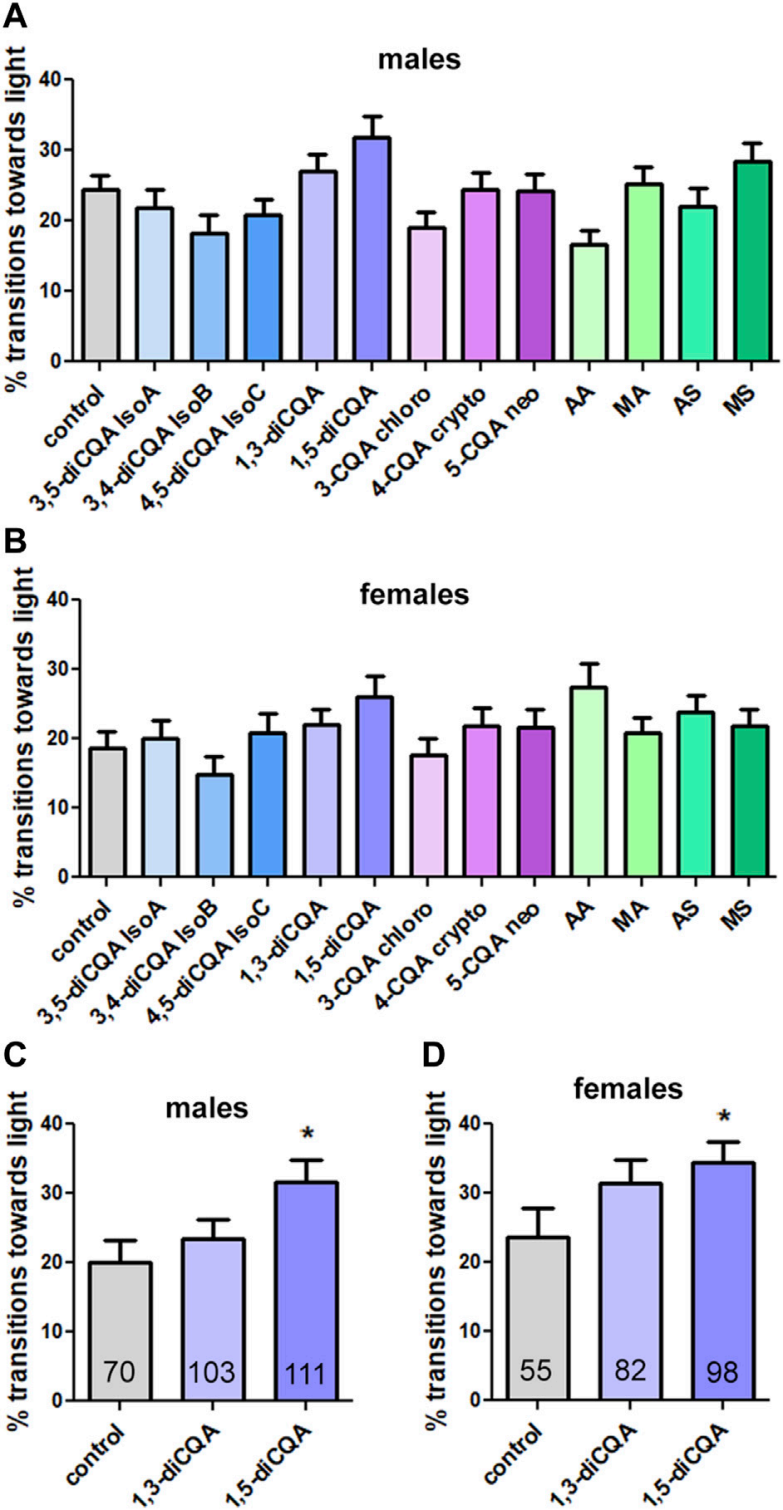
1,5-diCQA can improve the performance of male and female flies in the phototaxis assay. **(A, B)** Testing single compounds in one experiment did not result in significant effects of any compound in males **(A)** or females **(B)** although some improved the performance non significantly. **(C, D)** Testing 1,3-diCQA and 1,5-diCQA in a separate experiment *versus* vehicle controls (10% ethanol) resulted in significant improvement by 1,5-diCQA in males and females. The single compounds were given at concentrations equivalent to 10 mg/mL CAW. All flies were 42 days old and were tested in groups of 10–15 flies and separated by experimental group and sex. For A, B the number of tested flies was between 92 and 188 and for B the number of tested flies is indicated and in the bars. Error bars represent SEMs. Kruskal–Wallis tests for comparing multiple groups with post Dunn’s post-tests to compare pairs were used to determine significances between control and treatment groups. * = *p* < 0.05.

The results with the mixes suggested that both groups contained biologically active compounds and we therefore tested single CQAs and TTs at concentrations equivalent to their presence in CAW (10 mg/mL). However, none of them had a significant effect on phototaxis in males (Figure 5A) or females (Figure 5B) when testing all of them individually in one experiment and applying the appropriate Bonferroni correction for multiple groups. Nevertheless, some compounds improved the performance non-significantly. One of them was 1,5-diCQA, which increased the transitions towards light, in both males and females, and 1,3-diCQA which increased them in males. We therefore tested these compounds in a separate experiment. Comparing them to the included controls, 1,5-diCQA resulted in a significant improvement in males and females (Figures 5C, D), confirming that this CQA has biological activity in our assay. However, 1,5-diCQA was less protective than the CQA mix or CAW with increasing the locomotion score to about 35% in contrast to the CQA mix and CAW which increased it to about 60%.

## Discussion

CA has traditionally been used to improve memory and cognition enhancing effects have been described in aged animals and in a small clinical trial of healthy elderly (Wattanathorn et al., 2008b; Gray et al., 2016; Gray et al., 2018a; Sun et al., 2020). Furthermore, we and others have shown neuroprotective effects in animal models of Parkinson’s disease and Alzheimer’s disease (Haleagrahara and Ponnusamy, 2010; Soumyanath et al., 2012; Matthews et al., 2019; Zweig et al., 2021c). The present study explored the roles of TT and CQA components in activities of CAW related to neuronal health and function, using *in vitro* cell culture and *Drosophila* models.

Triterpenes (TTs) and caffeoylquinic acids (CQAs) are specialized metabolites known to occur in CA (Long et al., 2012; Yang et al., 2023a) and TTs, in particular, have been described as active compounds in neuroprotection by this herb (Gray et al., 2018a; Sabaragamuwa et al., 2018; Sun et al., 2020). Our results further support a role of TTs in promoting neuronal health because treating primary neurons with the TT mix increased dendritic complexity. Furthermore, it improved the performance of *Drosophila* in the fast phototaxis assay, as did CAW. However, in both models the CQA mix also had biological activity. Although CQAs have not received as much attention as the TTs as biological active compounds of CA, several CQAs could protect MC65 and SH-SY5Y neuroblastoma cells from Aβ-induced cell death (Gray et al., 2014). A CQA mix has also been shown to improve the memory in a mouse model of Alzheimer’s disease while a TT mix had no significant effect (Matthews et al., 2020). Due to both the TT and CQA mix having a positive effect in our experiments, we anticipated that combining both mixes might even have a stronger effect. However, this actually resulted in reduced dendritic arborizations and it did not increase the effect in *Drosophila* when given at concentrations equivalent to CAW. Reducing the levels to 50% to prevent a possible “ceiling effect” also did not show a stronger activity when combining the TTs and CQAs but instead it prevented the positive effect of the TT mix alone. A similar result was previously obtained when testing CAW and the mixes in a *Drosophila* model of depression (Holvoet et al., 2023a). Using this model, we showed that treating the flies with 10 mg/mL CAW significantly reduced the effects of chronic stress on motivation and anhedonia, which are depression-associated symptoms. Testing the mixes in the depression model showed that in that case the CQA mix was more protective than the TT mix and combining both mixes actually reduced the protective effect of the CQA mix. That combining both mixes reduced or even prevented the protective activity was surprising because the mixes contained the TT and CQA compounds at the same levels as in CAW. However CAW did increase arborization and provided resilience in the phototaxis test, as well as in the depression model as previously published (Holvoet et al., 2023a). Therefore, this indicates that the negative impact the TTs and CQAs have on each other may be overcome by additional compounds present in CAW; however additional studies are needed to address this issue.

Testing individual compounds confirmed positive effects of TTs on dendritic arborization, with MA and MS having the strongest effect. In contrast, none of them had a protective function in the *Drosophila* model at CAW equivalent concentrations. This suggests that, although a single compound can promote neuronal complexity, to provide resilience against the age-related decline in phototaxis a combination of TTs is needed. Six of the eight single CQAs also promoted dendritic arborization, with 5-CQA and IsoA being the exception. In the phototaxis test only 1,5-diCQA was protective and none of the other single compounds were, again indicating that a mix of the CQAs is more effective than a single compound. We previously showed that 1,5-diCQA (and 3-CQA) stimulated dendritic arborization of hippocampal neurons derived from wild type and the Tg2576 mouse Alzheimer’s disease model (Gray, Zweig et al., 2017). This indicates that 1,5-diCQA might be an important compound for the biological activity of CAW. However, this compound is the most abundant CQA in the tested batch of CAW. Since the concentration of each compound tested was based on its content in CAW, this may account for why only this compound was active. Several findings suggest that 1,5-diCQA may act by improving oxidative stress responses. Using neuroblastoma cells, 1,5-diCQA was shown to induce the expression of mitochondrial genes and it improved mitochondrial function (Gray et al., 2015). Reducing oxidative stress may also play part in the protective function of 1,5-diCQA in inhibiting apoptosis in cultured human corneal epithelia cells although this treatment also reduced inflammatory responses (Yoon et al., 2023). That 1,5-diCQA (and possibly to a certain degree other diCQAs) acts *via* reducing oxidative stress is also supported by the finding that the CQA mix protected *sniffer* mutant flies from phototaxis deficits while the TT mix did not (Cabey et al., 2022). The *sniffer* gene is a mutation in a carbonyl reductase that causes high oxidative stress and neurodegeneration in addition to deficits in locomotion (Botella et al., 2004). As shown in our cell culture model, some mono-CQAs (3-CQA and 4-CQA) also increased dendritic arborization, confirming previous studies showing that 3-CQA improved neuronal complexity of cultured hippocampal neurons from wild type and Tg2576 mice (Gray, Zweig et al., 2017). Although 3-CQA had no protective effect on the age-related decline in phototaxis, mono-CQAs and specifically 3-CQA did provide resilience in the *Drosophila* stress model (Holvoet et al., 2023a). It has been shown that 3-CQA, like 1,5-diCQA, has antioxidant and anti-inflammatory effects (Naveed et al., 2018; Alcazar Magana et al., 2020) but the difference in activity in the various assays suggests that they may not act in the same way. Whether this is due to acting on different targets or functioning in different cells or cellular compartments requires future studies. Lastly, our studies confirm the biological activity of TTs but again the effects are depending on the assay. Here, we show that the TT mix improves both, dendritic arborization and phototaxis but the single compounds only had an effect on dendritic arborization. Furthermore, the TT mix only partially protected from depression-induced phenotypes and it has no effect in the *sniffer* model (Cabey et al., 2022; Holvoet et al., 2023a). TTs have been ascribed anti-inflammatory functions and they can promote proliferation and migration (Agra et al., 2015; RdS et al., 2022). Activating pathways connected to proliferation and migration may be beneficial for dendritic arborization and anti-inflammatory properties may be beneficial for age-induced neuroinflammation, thereby improving locomotion. However, promoting these pathways may not be beneficial in the depression assays, which are done in young flies, or in phenotypes caused by oxidative stress as in the *sniffer* mutant.

Taken together, these results clearly demonstrate a role for both the CQAs and TTs in the neurotropic effects of CAW. When CA is sold as a dietary supplement, standardization, if reported, tends to focus on the triterpene content. Our findings show that studies are warranted to establish optimum levels of both the triterpene compounds and the CQA compounds in CA as a parameter for standardization.

## Data Availability

The original contributions presented in the study are included in the article/Supplementary Material, further inquiries can be directed to the corresponding author.
