# Supplementary material for: Centella asiatica and its caffeoylquinic acid and triterpene constituents increase dendritic arborization of mouse primary hippocampal neurons and improve age-related locomotion deficits in Drosophila
Source: Front Aging. 2024 Jul 11;5:1374905. doi: 10.3389/fragi.2024.1374905 (PMC11269084; doi:10.3389/fragi.2024.1374905)
Supplement: Supplementary file 1 [file Table1.docx]

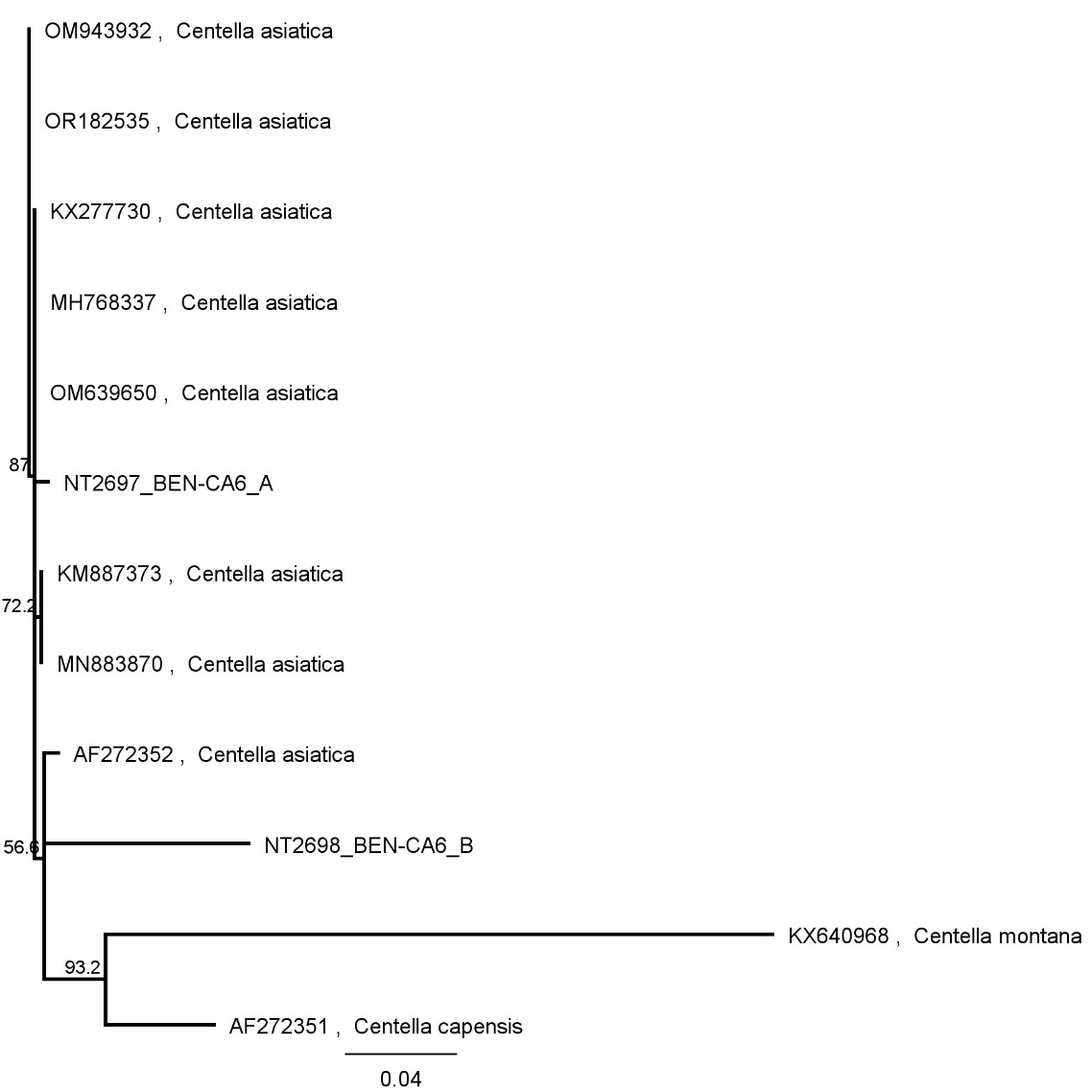


Supplementary Figure 1: Neighbor-joining tree with 500 bootstraps using the Tamura-Nei genetic distance model without an outgroup.
