# Supplementary figures and images for: Centella asiatica and its caffeoylquinic acid and triterpene constituents increase dendritic arborization of mouse primary hippocampal neurons and improve age-related locomotion deficits in Drosophila
Source: Front Aging. 2024 Jul 11;5:1374905. doi: 10.3389/fragi.2024.1374905 (PMC11269084; doi:10.3389/fragi.2024.1374905)

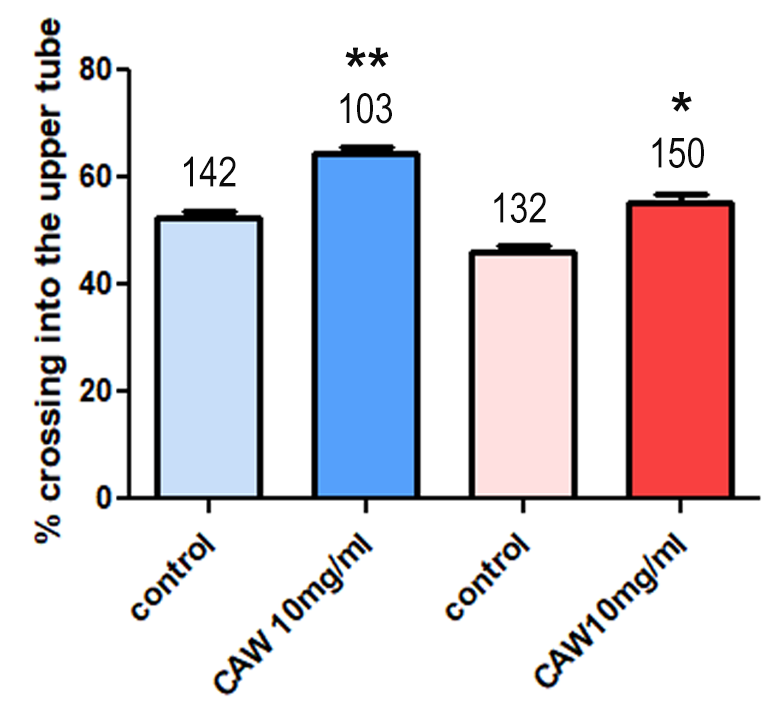

Supplement: Supplementary file 2 [file Image1.tif]
